# Anxiety disorders among youth with substance use and associated factors in Northwest Ethiopia: A community-based study

**DOI:** 10.1371/journal.pone.0300927

**Published:** 2024-03-21

**Authors:** Mamaru Melkam, Demeke Demilew, Tilahun Kassew, Biruk Fanta, Sewbesew Yitayih, Kassahun Alemu, Yassin Muhammed, Berhanie Getnet, Eden Abetu, Gebrekidan Ewnetu Tarekeg, Mohammed Oumer, Goshu Nenko

**Affiliations:** 1 Department of Psychiatry, College of Medicine and Health Science, University of Gondar, Gondar, Ethiopia; 2 Department of Biostatistics & Epidemiology, College of Medicine and Health Science, University of Gondar, Gondar, Ethiopia; 3 Department of Psychology, College of Social Science and Humanities, University of Gondar, Gondar, Ethiopia; 4 Department of Clinical Pharmacy, College of Medicine and Health Science, University of Gondar, Gondar, Ethiopia; 5 Department of Human Anatomy, College of Medicine and Health Science, University of Gondar, Gondar, Ethiopia; City University of New York, UNITED STATES

## Abstract

**Introduction:**

Anxiety disorder is an unpleasant emotional feeling with symptoms related to psychological and autonomic symptoms such as headache, perspiration, palpitations, dizziness, and stomach discomfort. The use of substances become a worldwide problem among youth which brings situation that leads to serious social and health-related problems. Anxiety disorders with substance use have a huge impact on their high prevalence, therapeutic issues, and poor prognosis on clinical effects. Although the prevalence of anxiety disorders is significant among young people who use substances, limited studies were conducted. Therefore, this study revealed the burden of anxiety disorders among youth with substance use and associated factors in Ethiopia.

**Method:**

A community-based multi-stage with a simple random sampling technique was conducted. A total of 372 substance users study participants were recruited for this study. Alcohol, Smoking, and Substance Involvement Screening Tests, Depression Anxiety Stress Scales, and other tools were used to assess anxiety disorders with substance use and associated factors. Data were entered into Epi-data version 4.6, and exported to SPSS version 20 for further analysis. Bi-variables logistic regression analysis was employed to identify variables with a p-value of < 0.2 and associated factors were determined in multi-variables logistic regression analysis with a p-value < 0.05 with AOR and CI.

**Results:**

From a total of 372 respondents the overall prevalence of anxiety disorders with substance use was 48.1%. Male sex [AOR = 1.99; 95% CI: (1.01–3.93)], low educational status of the father [AOR = 6.38 95%CI: (1.50–7.08)], and the presence of stress [AOR = 2.48; 95% CI: 2.48(2.43–4.40)] were significantly associated factors with anxiety disorders with substances use.

**Conclusions and recommendations:**

The prevalence of anxiety disorders with substance use was 48.1% therefore; it is recommended that the zonal administration give collaborative work with the health bureau and facilitate awareness creation about the impact of substance abuse. Clinicians are recommended to mitigate anxiety disorders with substance use to get a good prognosis for clients with controlling their stress.

## Introduction

Diagnostic and Statistical Manual of Mental Disorders (DSM-5) identifies anxiety disorders as the induction of clinically prominent fear, excessive worry, and panic symptoms with unknown cause or after taking substances or medications at the time of intoxication and withdrawal [1]. Anxiety disorders are characterized by a diffuse, unpleasant, vague sense of apprehension that is frequently accompanied by autonomic symptoms like headache, perspiration, palpitations, tightness in the chest, mild stomach discomfort, and restlessness [2,3]. Psychoactive substances change the function and the structure of the brain by altering the activity of specific pathways in the nervous system which can lead to anxiety disorders [4]. Studies showed that the use of substances with anxiety disorders can have a reciprocal relationship [5]. Almost all of the types of substances in DSM-5 are recognized to lead to anxiety disorders at the time of intoxication, withdrawal, or both [6]. The study showed that specific phobia (17.1%) was the most prevalent lifetime anxiety disorder followed by social anxiety disorder (10.7%) and generalized anxiety disorder (9.2%) the prevalences related to the use of a substance [7].

The effect of substance use and its effect on anxiety disorders is a major problem globally, particularly among youth [8]. Substance use and its impact on the neurotransmitter can make them vulnerable to other organic changes that have clinical signs and symptoms similar to anxiety disorders [9]. The particular pathway anxiety disorders that happen in the central nervous system varies based on the type of substances [10]. Determining the occurrence of anxiety disorders unrelated to substance use is crucial because substance use has complicated physiological and psychological effects that make it challenging to distinguish from the symptoms of anxiety disorders [3,11]. Worldwide reports have shown, that the consumption of tobacco is the major cause of death and disability related to anxiety disorders [12]. As the study indicates two out of every three substance users particularly alcohol users, the co-occurrence of anxiety disorders during some time in their life is common [13]. Even if the use of substances becomes high in the general population recent surveys report that the consumption of youth particularly alcohol is increasing which might result in anxiety disorders [14,15].

Alcohol is the most consumed substance in the community 7.6% of the global burden of disease and injury was attributable to alcohol consumption among men and 1.4% to consumption among women with anxiety disorders, including alcohol use was 36.4% [4]. Substances relatively commonly consumed in the general population within one year indicated a prevalence of 11% and ever use in life was a prevalence of 16.6% [16]. The prevalence of anxiety disorders ranges from 15% to 26% among substance users [11,17]. Many studies showed a high rate of anxiety disorders in the consumption of substances especially the use of alcohol and cigarette substances [11,18,19]. The comorbidity prevalence of anxiety disorders with substance use among youth was 67%. Other studies conducted among adolescents also revealed the prevalence of anxiety disorders with substance use was 42% [20] and 21% [18].

The association between anxiety disorders and substance use might be the ability of the substances to temporarily relieve the symptoms of anxiety disorders and the risk that substance users typically cause at the time of intoxication or withdrawal state [21]. Some factors were, associated with anxiety disorders with substance use in former studies incorporating; age, educational status of the respondent, sex, parental educational status, occupation, the presence of stress, absence of social supports, parental substance use, and peer use of a substance [22–26] The co-occurrence of anxiety disorders with substance use had subsequent problems are frequently exacerbated and become more complex, making it more difficult to manage and treat the afflicted persons, particularly among youth in developing countries [27].useThe co-occurrence of anxiety disorders and substance use has a huge problem related to their symptomatic presentations, clinical, policymaker, and treatment aspects [28]. Large-scale epidemiological research is not capable of identifying a relationship between anxiety disorders and substance use. Despite its burden among youth, studies show that anxiety disorders with substance use among the youth population are limited. Our study can provide a direction for policymakers and clinicians to facilitate the therapeutic duration of these problems particularly in low- and middle-income countries including Ethiopia. Therefore, this study revealed the prevalence of anxiety disorder among youth with substance use and its associated factors in Ethiopia.

## Methods and materials

### Study period and setting

A multi-stage cross-sectional study was conducted in the Central Gondar Zone, in Northwest Ethiopia. Central Gondar Zone is located in Amhara regional state, Northwest of Ethiopia which occupies 21791.83 KM. Central Gondar is a recently established zone formerly inside of the North Gondar zone. In this zone, there are a total of 16 districts and 442 kebeles. The central Gondar zone has a population of 2,642,138, according to the population statistics estimate of 2012 E.C. Young people (15 to 24 years old) made up 575 656 (21.79%) of the total population. The number of youths is 286,385 (males) and 289,271 (females), respectively.

### Source and study population

All youth who live in the Central Gondar Zone with the age of 15 to 24 were considered as the source of the population. All youth in the Central Gondar Zone who have used substances and live in the selected Kebeles of the Zone were considered as a study population.

### Eligibility criteria

All youth aged from 15 to 24 years old were included in the study and youth who were difficult to communicate due to severe medical illness were excluded from the study.

### Sample size and sampling technique

The sampling methods had two stages sampling, the first one was calculated for substance use and the second stage was to determine the anxiety disorders with substance use among substance users only. Initially, the required sample for this study was determined by using both single population proportion formulas and double population proportion formulas which were calculated by using Epi-Info software. The higher sample calculated by single population proportion formulas was taken. A geographical cluster was used based on each Woreda [6], and then down to Kebele from these Woredas, to provide an equal chance for selection by simple random sampling. The numbers of participants at each randomly selected Kebele were allocated proportionally based on the size of the youth. Finally, from the randomly selected Kebeles, participants were recruited from the Ketenas under Kebele with a cluster sampling technique which was surveyed randomly until a proportional number was reached.

An additional sampling technique was taken from individuals who used one or more substances in the last three months from the whole sampled youth, to consider the development of anxiety disorders from substance users. Three hundred seventy-two (372) participants were recruited samples as current substance users that were considered for the analysis of anxiety disorders with substance use.

### Operational definitions

**Anxiety disorder with substance use:** the presence of anxiety based on DASS-21 among substance users means the occurrence of anxiety disorders from the substance users (alcohol, chat, and cigarette) in this study [29–31].

**Youth:** is used to denote the late adolescents and young adults with the age of 15–24 [32].

**Ever substance use:** means based on ASSIST individuals who use at least one of specified common substance in their life.

**Current substance use:** means based on ASSIST individuals who use at least one of the specified common substances in the last three months [29]

**Anxiety:** is defined based on the DASS-21 anxiety subscale, individuals who scored greater than or equal to 15 are considered as having anxiety.

**Stress:** is defined based on the DASS-21 stress subscale, individuals who scored greater than or equal to 26 are considered as having stress.

**Perceived social support:** The total mean score across all MSPSS-12 items, any mean scale score ranging from 1 to 2.9 could be considered low support; a score of 3 to 5 could be considered moderate support; a score from 5.1 to 7 could be considered high support.

### Study variables

#### Dependent variables

✓ Anxiety disorders with substance use.

#### Independent variables

✓ **Personal characteristics:** Gender, age, religion, marital status, educational status, occupation, residency, current living arrangement.

✓ **Family-related variable**s: family size, father’s/mother’s level of education, occupational status of father/ mother, biological parents alive, having a family member using a substance, the recent loss of loved ones, having a friend using a substance.

✓ **Psycho-social variables:** Perceived social support, level of psychological stress.

### Data collection materials and procedure

The **first part** of the data was a socio-demographic questionnaire for basic demographic and family-related characteristics of participants.

The **second part** of the data was the Alcohol, Smoking, and Substance Involvement Screening Test (ASSIST) questionnaire that screens for all levels of the problem or risky substance use in adults. The ASSIST consists of eight questions covering tobacco, alcohol, cannabis, cocaine, amphetamine-type stimulants (including ecstasy) inhalants, sedatives, hallucinogens, opioids, and ’other drugs. A risk score was provided for each substance, and scores are grouped into ’low risk’, ’moderate risk’, or ’high risk’. Then the level of risk of health and other problems from the current pattern of use was rated by the sum across questions 2–7 for each drug category separately. The WHO recommended ASSIST cut-off scores for conventional risk levels (low, moderate, high) are as follows: for alcoholic beverages: 0–10 rated as low risk; 11–26 moderate; 27+ as high risk experiencing severe problems as a result of the current pattern of use and are likely to be dependent. For other drug categories separately scores 0–3, 4–26, and 27+ indicate a low, moderate, and high risk of experiencing health and other problems as a result of the current pattern use of a drug.

The **third part** Depression, Anxiety, and Stress Scale-21 Items (DASS-21) was a set of three self-report scales designed to measure the emotional states of depression, anxiety, and stress. But in this study, the two which means anxiety and stress were used. Each of the DASS-21 scales contains 7 items, divided into subscales with similar content. It assesses difficulty relaxing, nervous arousal, being easily upset/agitated, irritable/over-reactive, and impatient. Scores for anxiety and stress are calculated by summing the scores for the relevant items [30]. The responses for each statement scored on a Likert scale from 0 to 3 which indicates how much the statement applied to you over the past week. DASS-21 will need to be multiplied by 2 to calculate the final score.

The recommended cut-off scores for conventional severity labels (normal, moderate, severe) are as follows: Anxiety: 0–7 normal; 8–9 mild; 10–14 moderate and 15 and above severe. Stress: 0–14, 15–18, 18–25, and 26 and above are considered normal, mild, moderate, and severe respectively. DASS-21 is based on a dimensional rather than a categorical conception of psychological disorder. The DASS-21, therefore, has no direct implications for the allocation of patients to discrete diagnostic categories postulated in classificatory systems such as the DSM and ICD. The sensitivity and specificity of the DASS-21 tool were 69.2% and 75.5% respectively. The overall Cronbach alpha of the DASS-21 was 0.74 with ordinal alpha of 0.83, 0.74, and 0.87 for DASS depression, anxiety, and stress respectively [31].

The **fourth part** of the questionnaire was the Multidimensional Scale of Seen Social Support (MSPSS), which is a 12-item scale to gauge how family, friends, and a significant other are perceived to be supporting you. The scale has a total of 12 elements, 4 of which are for each subscale [33]. The response to each item is given on a Likert scale, with 1 being very strongly disagree and 7 being agree. Following is how the mean score for subscales is determined:

Significant Other Subscale: Sum across items 1, 2, 5, & 10, and then divide by 4. Family Subscale: Sum across items 3, 4, 8, & 11, and then divide by 4. Friends Subscale: Sum across items 6, 7, 9, & 12, and then divide by 4. Total Scale: Sum across all 12 items, then divide by 12. In this approach any mean scale score ranging from 1 to 2.9 could be considered low support; a score of 3 to 5 could be considered moderate support; a score from 5.1 to 7 could be considered high support. Across many studies, the MSPSS has been shown to have good internal and test-retest reliability, good validity, and a fairly stable factorial structure [33,34].

### Data quality assurance

The questionnaire first was prepared in the English language; then it was translated by a bilingual translator into the Amharic language. To check its consistency with its English version, it was retranslated to the original version. Fifteen data collectors and five supervisors were selected, and training was given for 8 hours within two consecutive days. The supervisors regularly evaluated the accuracy and consistency of the data. Before performing the survey outside a predetermined study area, 5% of youth completed a pre-test for all structured questionnaires.

### Intervention strategies for our outcome variables

In this study, participants who were diagnosed with severe anxiety disorders were linked to health facilities for further evaluation and treatment. Participants who were found to have anxiety disorders with substance use were linked to health facilities for the evaluation of their disorders. Addictive behaviors did not change abruptly but through a series of stages: pre-contemplation, contemplation, preparation, action, and maintenance. Participants who were in different stages were linked to the psychiatrists to explore and strengthen their motivation, develop relapse prevention strategies, and address underlying predispositions through individual therapy, family therapy, and pharmacotherapy.

### Data analysis procedure

The data entry was performed using the statistical program Epi-Data 4.6 version and then exported into SPSS version 20 for analysis. The data distributions were summarized by descriptive statistics. The bi-variables logistic regression analysis was carried out to analyze the important associated factors with anxiety disorders. In the bi-variable analysis, statistically significant variables by p-value <0.2 to recruit candidate variables for multi-variable analysis. Adjusted odds ratio with a 95% confidence interval was used to declare statistically significant variables based on p-value <0.05 in the multivariable logistic regression model. The basic assumptions and model fitness of the statistical analysis model used in this study was 65.10 on the Hosmer and Lemeshow test. The absence of collinearity among independent variables was determined in normal circumstances it must be less than 10, therefore, there is no co-linearity with VIF less than one in this research.

### Ethical approval

Ethical clearance was obtained from the University of Gondar Institutional Review Board (IRB) with reference number (V/P/RCS/05/551/2020). administrative approval was received from the zone authorities before the data collection. Participants were informed about the study which had no negative impact on their lives, their confidentiality was maintained, and written informed consent and assent were obtained from the youth and their parents before the data collection period, respectively. Participants were informed that they had a right to refuse participation at any time they wanted and had also the right to ask about concerns about the study. The privacy of the participants and the confidentiality of the data was maintained.

## Results

### Socio-demographic factors

A total of 372 study participants were current substance users for the last three months. Among those study participants, 281(75.5%) were male and 91 (24.5%) were female. The mean age of the participants was 20.64 with a standard deviation of **±**2.60. The largest number of respondents were from the Orthodox religion 314(84.4%) and 255(68.5%) were from urban residences. Among this study participants, 312(83.9%) were married and more than half of the respondents 258(69.7%) had high school educations. Of the study participants, 216(57.9%) were students and 276(74.5%) were currently living alone. From this study 315(84.7%) of the participant both biological parents alive. Of the study participants, 153(41.3%) had family members who use alcohol or other substance. Of the respondents, 124(33.1%) lost their loved one recently in the past 6 months and 194(52%) had a friend who uses alcohol (Table 1).

**Table 1:** Socio-demographic characteristics of study participants in Northwest Ethiopia.

**Variables**	**Category**	**Frequency**	**Prevalence**
**Sex**	Male Female	281 91	75.5 24.5
**Age**	≤ 19 20–24 ≥ 25	137 229 6	36.8 61.6 1.6
**Religion**	Orthodox Muslim Others*	314 51 7	84.4 13.7 1.9
**Marital status**	Married Single Others**	312 53 7	83.9 14.2 1.9
**Education**	Discontinued 1^st^ school 2^nd^ school College/university	28 77 183 84	7.6 20.7 50 22.7
**Job**	Government employed Merchant Farmer Students Day laborer Others***	32 51 20 216 25 30	8.6 13.3 5.4 57.8 6.7 8.1
**Residency**	Urban Rural	255 117	68.5 31.5
**Living status**	With family Alone Others****	89 276 7	23.6 74.5 1.9
**Both parents alive**	Yes No	315 57	84.7 15.3
**Family alcohol use**	Yes No	153 219	41.3 58.7
Father educational status	Unable to read/write 1^st^ school 2^nd^ school College/university	179 118 30 45	47.9 31.8 8.1 12.2
Mother educational status	Unable to read/write 1^st^ school 2^nd^ school College/university	233 78 25 34	63 21.2 6.8 9.2
**Loss of a loved one**	Yes No	124 248	33.1 66.9
**Friends’ use of alcohol**	Yes No	194 178	52 48

Others* = protestant, catholic.

Others** = divorced, widowed.

Others*** = housewife, jobless.

Others**** = live with other relatives.

### Psychosocial factors

Among the participants 136(36.3%) had high social support, 185(50%) had moderate social support and 51(13.8%) had low social support. Of the study participants, 170(46%) had been exposed to moderate and high stress (Table 2).

**Table 2:** The psychological factors from the study participants in the central Gondar zone in Northwest Ethiopia.

Variables	Category	Frequency	Percentage
**Stress**	Normal Mild Moderate Sever	145 56 80 91	31.2 14.9 24.6 21.4
**Social support**	High Moderate Low	136 185 51	36.3 50 13.7

### Prevalence of anxiety disorders with substance use

**Fig 1:**
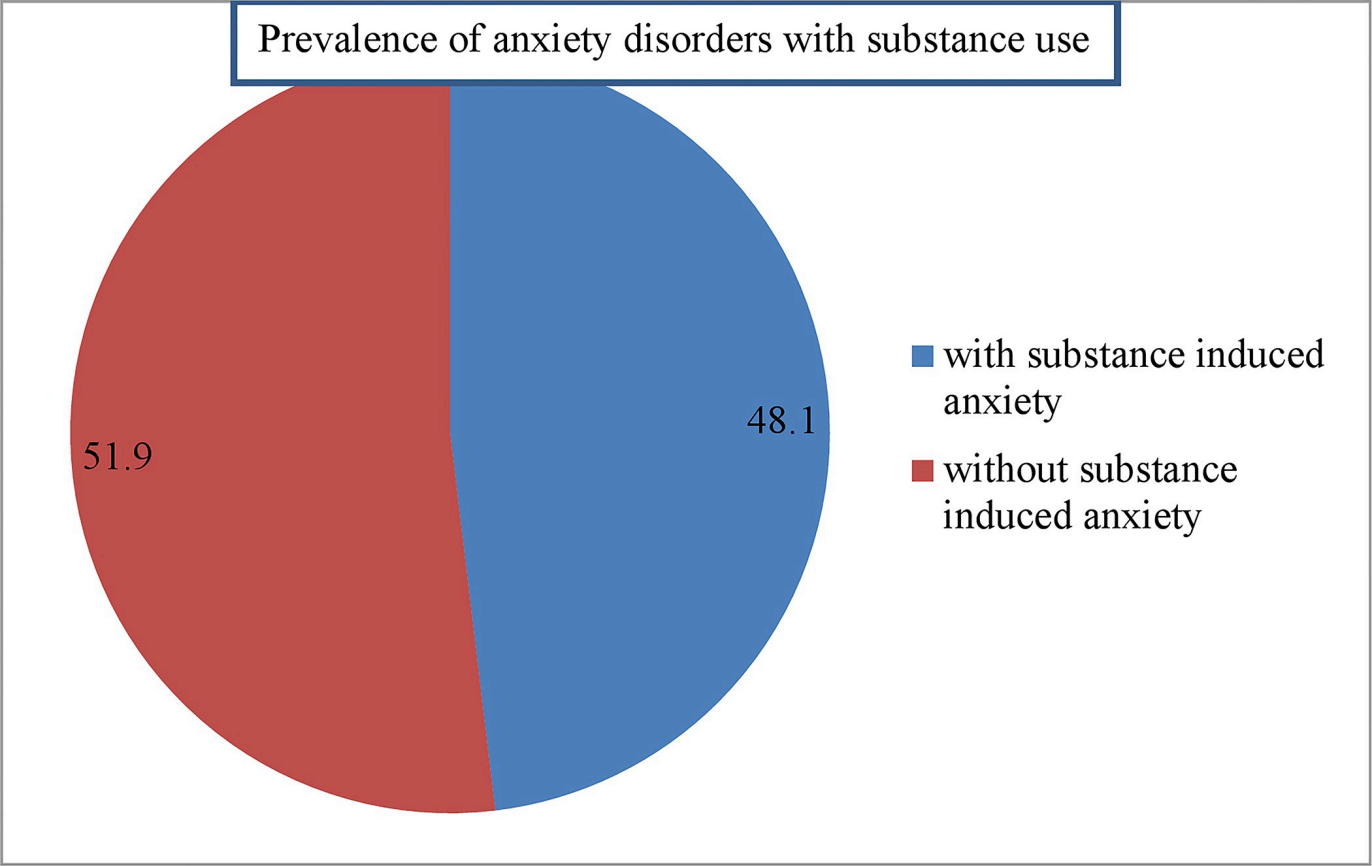
Schematic presentation of sample for selecting the study participants in the Gondar zone.

The prevalence of anxiety disorders with substance use among the study participants in the central Gondar zone of Northwest Ethiopia was 178 (48.1%) with a 95% CI; (42.99, 53.22) (Fig 1).

### Associated factors of anxiety disorders with substance use

From bi-variable logistic regression being male, religion low education states, family alcohol use, friend alcohol use, both parents’ loss, low educational status of mother, and father, low family support, and presence of severe stress were associated with anxiety disorder with substance use with a p-value of less than 0.2. From bi-variables and multi-variables logistic regression analysis male sex, low educational status of the father, and the presence of stress were significantly associated with a p-value of less than 0.05 with anxiety disorders with substance use.

Among the variables, male sex was 1.99 times more likely to have anxiety disorders with substance use when compared to female participants [AOR = 1.99; 95% CI: (1.01–3.93)].

The probability of low father education to develop anxiety disorders with substance use was 6.38 times more in having a father who was unable to read and write as compared to others with high educational status [AOR = 6.38 95%CI: (1.50–7.08)].

The odds of developing anxiety disorders with substance use were high risk among the respondents with stress as compared to the participants without stress [AOR = 2.48; 95% CI: 2.48(2.43–4.40)] (Table 3).

**Table 3:** Factors associated with anxiety disorders with substance use logistic regression analysis in Northwest Ethiopia.

Variables	Category	Sub-induced anxiety		COR with 95% CI	AOR with 95% CI
		Yes	No		
**sex**	Male Female	144 35	137 56	1.68(1.04–2.72) 1	1.99(1.01–3.93) 1
**Age**	≤ 19 20–24 ≥ 25	59 116 4	78 113 2	1 1.36(0.88–2.08) 2.64(0.47–14.9)	1 0.67(0.34–1.29) 0.71(0.78–6.42)
Education of participant	Discontinued 1^st^ school 2^nd^ school College/uni	20 41 75 43	8 38 106 41	2.38(0.94–6.01) 1,03(0.55–1.91) 0.67(0.40–1.13) 1	2.32(0.64–8.47) 0.93(0.35–2.46) 0.61(0.27–1.36) 1
Both parent alive	Yes No	147 32	168 25	1.46(0.83–2.58) 1	0.99(0.43–2.28) 1
Father occupation	Gov. employed Farmer Merchant Day laborer Retired Other	39 112 22 9 5 6	17 111 32 6 5 8	1 2.27(1.21–4.25) 3.33(1.51–7.33) 1.52(0.47–4.97) 2.29(0.59–8.97) 3.06(0.29–10.17)	1 0.93(0.32–2.73) 1.98(0.60–6.47) 0.32(0.06–1.67) 1.01(0.15–6.56) 3.68(0.58–24.16)
Father education	Not read/write 1^st^ school 2^nd^ school College/uni	97 54 17 12	80 64 14 33	3.64(1.73–7.65) 2.53(1.17–5.48) 3.42(1.27–9.22) 1	6.38(1.50–7.08) 6.06(1.48–24.78) 6.63(1.39–31.53) 1
Mother education	Not read/write 1^st^ school 2^nd^ school College/uni	126 30 11 12	106 48 15 23	2.48(1.16–5.33) 1.30(0.56–3.06) 1.64(0.57–4.77) 1	0.43(0.10–1.84) 0.43(0.10–1.90) 0.23(0.04–1.32) 1
Family alcohol use	Yes No	61 119	93 99	0.54(0.36–0.82) 1	1.25(0.63–2.46) 1
Friend’s alcohol use	Yes No	110 69	83 110	2.17(0.36–0.82) 1	0.66(0.33–1.35) 1
Loss of loved one	Yes No	71 107	51 141	1.84(1.18–2.85) 1	1.39(0.73–2.66) 1
**Family support**	Low Moderate High	24 69 101	25 95 58	1.87(0.98–3.59) 2.37(1.52–3.72) 1	1.61(0.58–4.47) 1.92(1.00–3.71) 1
**Stress**	Normal Mild Moderate Sever	130 24 29 10	15 32 63 69	1 1.20(2.26–5.80) 9.50(3.72–9.09) 5.80(5.51–14.16)	1 6.91(0.09–4.36) 5.90(0.92–6.86) 2.48(2.43–4.40)

Uni = University.

Gov. = Government.

## Discussions

The prevalence of anxiety disorders with substance use among the study participants was 48.1% with a 95% CI (42.99,53.22). This topic is not much studied yet as a result there are no publications that are in line with our study. The type of substance that is consumed among youth may influence the prevalence of anxiety disorders with substance use, and the type of anxiety disorders that occur also vary depending on the substances used and it may develop during intoxication, withdrawal state, or both [35]. The prevalence of this finding was high as compared to other studies conducted in Spain 42% [20] and Iran 21% [18]. The major discrepancy between our study and the Spain and Iran studies might be the measurement tools used were diagnostic DSM-IV but in our study, we have used the DASS-21 screening tool [18,20]. Another reason could be the effect of participant socio-demographical differences, for instance, the low educational level of the parents was a risk in this study which might have had high educational status in other studies carried out in Spain and Iran [18,20].

The finding of this study was lower as compared to other studies conducted in South Carolina 67% [36]. The discrepancy might result from differences in the study participants; that is, the current study was conducted among community-based students, whereas the prior study involved students who experienced higher levels of stress connected to their schooling [36]. The other reason might be the effect of high socio-demographic differences being white becomes more involved in substance use than black which increased as compared to this study area [26]. The other reason for the discrepancy might be the effect of high social interaction in the Ethiopian community that helps in coping with stress and anxiety [37].

The overall result indicated the factors associated with anxiety disorders with substance use were male sex as compared to female. The possible reason for the associations could be due to the high consumption of substances as evidenced in another research conducted in the United States and Ethiopia [22–24]. Even though most anxiety disorders were more risk among females, the effect of using substances was higher among males which led to an increase in the occurrence of anxiety [38]. Being a man increases one’s exposure to peer pressure to take drugs, one’s involvement in recreational activities, and one’s parental inhibition, which all increase the chance of developing anxiety problems brought on by drugs [39]. The other possible reason for this association might be the effect of the culture of females not using substances as compared to males that finally protect females from anxiety disorders [35].

The other determinant factor for the anxiety disorders with substance use was the low educational status of the participant’s father this might be because of the dis-inhibition of the parent that leads to the use of substances that results in the development of anxiety disorders. The low educational status of the father related to adolescent use of the substance was associated with adolescents bringing lower levels of some protective effect or higher levels of risk factors [38]. This may be a result of the parents’ belief that their freedom is essential to a happy family and the bad model of their father particularly substance abdicated leads them to have substances that cause anxiety.

The presence of stress among the participants was another important indicator for the development of anxiety disorders with substance use this might be the impact of the poor coping mechanism [25]. Even though the temporal association was not clear due to the nature cross-section study design the participant might use substances to relieve the existing stress which finally leads to anxiety disorders with substance use [25]. The other possible reason could be the effect of substance use to cope with stress as a negative coping mechanism [40]. The nature of the stress, the person’s psychological defense, and the coping mechanisms of the individual might be the determinant to perceive the event as stressful [41,42].

## Limitation

Even though this study is crucial to be the first study in Ethiopia related to anxiety disorders with substance use it has its limitations. The nature of the cross-sectional study design can’t show the cause-and-effect relationship. The other limitation could be social desirability bias being a substance abuser.

## Conclusions and recommendations

Nearly half of the study participants or youth had anxiety disorders with substance use. The co-occurrence of anxiety use with substance use was determined by factors like educational level, being male, and the presence of stress. Therefore, the collaborative reassessment by using diagnostic criteria and giving pharmacological and/or psychotherapy for those who need support. To reduce both the prevalence of substance use and anxiety disorders with substance the determinant factors should be controlled or minimized by integrative work with different stakeholders. Initially, it is highly recommended that youths reduce the consumption of substances and use entertainment and sports to substitute for their addiction. In order to support the younger generation, clinicians should collaborate with the zone administration to provide assessment and therapies for those who develop anxiety problems.
